# Patient Experiences of Digital Technology Use in Interstitial Lung Disease (PRODIGY-ILD Study): Qualitative Study Using Reflexive Thematic Analysis

**DOI:** 10.2196/91533

**Published:** 2026-08-25

**Authors:** Emer Gunne, Alessandro Franciosi, Cormac McCarthy, Michael P Keane, Peter Doran, Sinead Holden

**Affiliations:** 1 Clinical Research Centre, School of Medicine, University College Dublin, Elm Park, Dublin, 4, D04T6F4, Ireland, 353 (1) 2214000; 2 St. Vincent's University Hospital, Dublin, Leinster, Ireland; 3 Belfield, College of Health and Agricultural Science, University College Dublin, Dublin, Leinster, Ireland; 4 Belfield, School of Public Health, Physiotherapy & Sports Science, University College Dublin, Dublin, Leinster, Ireland

**Keywords:** digital technology, interstitial lung disease, remote monitoring, clinical trials, qualitative thematic analysis

## Abstract

**Background:**

Digital health technology enables collection of continuous physiological and behavioral data from participants in clinical trials. This supports hybrid trial designs, potentially reducing clinic visits and participant burden for patient monitoring. Interstitial lung disease (ILD) is characterized by an unpredictable clinical course, creating a need for new treatments and more sensitive approaches to assessing treatment effectiveness, disease progression, and clinically meaningful trial end points.

**Objective:**

This study aimed to explore the experiences of individuals with ILD using digital tools in a clinical study to inform digital health–enabled clinical research.

**Methods:**

This qualitative study was conducted within the PRODIGY-ILD (Predicting Outcomes using Digital Technology in Interstitial Lung Disease) cohort, a prospective observational study using wearable devices and electronic patient-reported outcome measures for a planned 3 years of longitudinal monitoring. Participants were recruited from a specialist outpatient ILD clinic. A topic guide was developed iteratively, and individual semistructured interviews were conducted remotely via Zoom (Zoom Video Communications, Inc) and/or telephone, audio-recorded, and transcribed. Data were analyzed using reflexive thematic analysis with NVivo software (Lumivero).

**Results:**

Fifteen of the final 20 participants recruited to the PRODIGY-ILD study consented and completed interviews. Four key themes were identified, highlighting how trust, digital literacy, participant-initiated engagement with data, and illness burden shape sustained participation in digital health–enabled clinical research: (1) trust and altruism override data concerns: confidence in researchers’ data handling and a desire to contribute enabled data sharing; (2) navigating digital tools: friction and flexibility: digital literacy, usability, and device compatibility varied, but participants were able to use workarounds to maintain engagement; (3) participant-initiated engagement with wearable data: participants moved from passive to active engagement, in many cases integrating devices into daily routines; and (4) life-limiting illness as a constraint on digital trial participation: managing symptoms and severe comorbidities reduces motivation and engagement with study technology.

**Conclusions:**

Despite participants’ motivations to contribute data to research, engagement was shaped by usability, participant-initiated engagement, and the constraints of living with chronic illness. There is a need for patient-centered design, tailored support, and flexible trial procedures to optimize adherence in digital health–enabled clinical research.

## Introduction

Interstitial lung diseases (ILDs) are a heterogeneous group of chronic respiratory conditions characterized by significant symptom burden and progressive declines in lung function typical of fibrotic phenotypes [1]. The clinical course of ILD is often unpredictable, with periods of stability punctuated by episodes of deterioration that can be difficult to identify early using traditional, clinic-based measures [1]. Recent studies on the feasibility and acceptability of home monitoring to provide real-world data for patients have been promising [2-9]. Advances in digital technology, including smartphone apps and wearables, offer opportunities to collect continuous and timely data on physical activity, symptoms, and physiological parameters to augment outpatient clinical data and to monitor participants in clinical trials [10].

Although digital monitoring in ILD has shown promise, a mixed methods study by Althobiani et al [11] suggested that patients may experience both reassurance and burden when engaging with self-management using digital monitoring, with engagement shaped by symptom severity, treatment effects, and uncertainty around how monitoring data are interpreted or acted upon clinically. However, existing studies have largely assessed the acceptability of digital monitoring using cross-sectional surveys [11] or short-term evaluations; little is known about how patients experience sustained engagement with these technologies over time. This study addresses this gap by examining the lived experience of participating in a longitudinal digital monitoring program for ILD, capturing how engagement evolves across differing clinical trajectories. Understanding how patients experience, perceive, and adapt to digital monitoring in real-world contexts is essential to ensure that these technologies are acceptable, feasible, and responsive to patient needs, facilitating sustained engagement and data quality.

PRODIGY-ILD [12] (Predicting Outcomes using Digital Technology in Interstitial Lung Disease) is a prospective cohort study designed to examine whether remotely collected electronic patient-reported outcome measures (ePROMs) and wearable-derived physiological data can identify early indicators of disease trajectory in people living with ILD, for a planned 3-year follow-up. In this study, participants completed regular symptom-based ePROMs, including weekly and monthly measures, and wore a smartwatch that passively captured behavioral and physiological data throughout follow-up, as described in the study protocol [12]. The primary quantitative analysis focus was on identifying candidate digital biomarkers and understanding temporal patterns in symptoms and physiology across the disease course. What remains underexplored is how participants live with, interpret, and adapt to digital monitoring over time, particularly when technologies are embedded within life-limiting illness. This qualitative substudy, nested within the PRODIGY-ILD study, aimed to explore participant experiences, attitudes, and perceptions of using the MyCap app (Vanderbilt University Medical Center), the companion app to REDCap (Vanderbilt University) research software for ePROMs and the Apple Watch wearable device, to inform the development of future digital health design for clinical trials [13,14].

## Methods

### Design and Theoretical Approach

#### Overview

This study was designed as a qualitative study with individual interviews. A qualitative design with individual interviews was selected to capture the depth, nuance, and lived experience of participants in a one-to-one setting, enabling a rich and contextualized understanding that cannot be fully represented through quantitative measures alone. The COREQ (Consolidated Criteria for Reporting Qualitative Research) [15] 32-item checklist (Checklist 1) informed the study design and was used for transparency and completeness of reporting interviews.

#### Positioning

This study was informed by an experiential critical realist stance [16], recognizing that while ILD and associated physiological changes represent real underlying phenomena, participants’ experiences of symptoms and digital health technologies are subjectively perceived and socially mediated. Knowledge was therefore understood as being generated through participants’ accounts of lived experience, coconstructed through interaction with the researcher and shaped by the analytical framework applied.

This epistemological position has important analytic implications, particularly given the researcher’s prior involvement in the wider PRODIGY-ILD study. Rather than treating prior relationships as a source of bias to be eliminated, they were understood as a contextual feature of the research process that could shape both data generation and interpretation. Familiarity between participants and the researcher may have influenced what participants chose to disclose, how experiences were framed, and the assumptions embedded within accounts.

Reflexive thematic analysis was therefore used, explicitly acknowledging the active role of the researcher in meaning-making. Reflexivity was operationalized through ongoing critical self-reflection throughout data collection and analysis, including the use of reflexive memo-writing to document assumptions, emotional responses, and evolving interpretations, and regular revisiting of analytic decisions to consider how the researcher’s positionality and prior knowledge may have shaped theme development. Themes are understood as analytic constructions rather than emergent truths, and interpretations were iteratively refined through engagement with the data and discussion within the research team, ensuring that themes remained grounded in participants’ accounts while recognizing the interpretive nature of qualitative analysis.

#### Development of Interview Guide

The interview guide was developed through a comprehensive process that drew upon existing literature, team discussions, and pilot testing with 2 participants. The approach was iterative, allowing for continual refinement to ensure the guide was both relevant to the study objectives and responsive to participant feedback. Key topic areas included onboarding, usability, perceived usefulness, impact on daily life, privacy concerns, and suggestions for improvement. The guide consisted of open-ended questions, supplemented with targeted prompts, to facilitate in-depth exploration of participant experiences. Further details of the interview template and question structure are provided in Multimedia Appendix 1.

#### Participant Recruitment and Sampling

Participants were recruited from the existing PRODIGY-ILD study cohort (n=20 final number onboarded between April 30 and November 22, 2024) attending the specialist ILD outpatient clinic. The inclusion criteria were as follows: (1) age ≥18 years, (2) diagnosis of idiopathic pulmonary fibrosis or progressive pulmonary fibrosis, and (3) ability to provide written informed consent. The exclusion criteria were as follows: (1) patients who are unwilling to wear a smart watch for the duration of the study and (2) cognitive impairment or inability to understand and follow instructions that would limit the patient’s understanding of the project or the measurement.

A purposive sampling approach was used, whereby individuals were invited to participate based on their direct experience of living with ILD and engagement with wearable and app use as part of the parent study. All eligible participants were sent a text message and a hard-copy patient information leaflet and informed consent form to inform participants of the qualitative substudy (Multimedia Appendix 2). Reasons for nonparticipation included death, withdrawal from the parent study, being too unwell, or personal reasons.

#### Data Collection

One-on-one semistructured interviews were conducted between July 16 and August 20, 2025, using the Zoom online platform (Zoom Video Communications, Inc) to conduct and record the semistructured interview using Zoom’s autotranscription function. Three participants were unable to log on to Zoom and were interviewed by phone, the audio of which was recorded and autotranscribed by Zoom. Patient A’s wife was present and Patient B’s son was present during the interviews; however, both answered all questions independently. Participants were interviewed once. Interviews lasted between 18 and 51 minutes and were recorded with permission. Notes were taken after each interview to document details and initial impressions. Transcripts were checked against the recordings for accuracy after each interview. All transcripts were anonymized according to COREQ. Member checking was not performed to reduce participation burden.

#### Data Analysis

Reflexive thematic analysis was conducted in line with Braun and Clarke’s 6-phase approach [17,18], using an inductive approach, allowing patterns of meaning to emerge from participants’ accounts, such that theme development was not constrained by predefined categories.

Thematic analysis proceeded through the following stages, once audiochecked anonymized transcripts were imported into NVivo qualitative data analysis software (Table 1).

**Table 1.** Practical workflow.

Step	Activity	Tool or platform
Interview and record	Semistructured interviews	Zoom and/or phone call
Transcription	Auto + manual cleaning	Word
Coding and theme generation	Manual and NVivo and Word	NVivo and Word
Reflection and reporting	Summarize and contextualize	Word and Word tables

Researchers familiarized themselves with the transcripts by reading them multiple times. They then generated initial codes, which included usability domains such as onboarding and reminders, as well as inductive codes that emerged directly from the data. These codes were subsequently grouped into candidate themes, which were reviewed and refined iteratively to ensure coherence and distinctiveness, reflecting the principles of reflexive thematic analysis. Analytic credibility was supported through the maintenance of a detailed audit trail documenting iterative theme development. Regular discussions among the research team provided a forum for reflexive dialogue, in which interpretations were questioned, alternative readings considered, and analytic decisions refined, recognizing that themes represent informed analytic judgment rather than objective consensus. Consideration was also given to the inclusion of deviant cases and contrasting viewpoints, with divergent perspectives actively sought and incorporated to ensure a comprehensive understanding of participants’ experiences.

Once the themes were finalized, they were defined and named, and the findings were reported with illustrative participant quotes to provide context and depth.

NVivo was used for parent and child coding; the coding structure is detailed in Multimedia Appendix 3. Themes are presented alongside tables of relevant participant quotes, and divergent perspectives are highlighted to reflect a range of experiences. The clinical trial context is also provided to aid the interpretation of themes within the broader study framework.

#### Research Team and Reflexivity

Interviews were conducted by EG pursuing a PhD in digital technology for clinical trials at the time of the study. All participants were known to the researcher through their enrollment in the PRODIGY-ILD study prior to the interviews. The interviewer introduced herself in the context of her research role and explained that the interviews sought to explore participants’ experiences and perspectives on wearable technology use and apps for patient-reported outcomes. Participants were aware of the overarching study aims but were not provided with the specific interview questions in advance.

This prior familiarity between the interviewer and participants may have fostered a sense of trust and openness, encouraging participants to share their honest experiences and perspectives more freely. Additionally, the interviewer’s background and expertise in clinical trials and digital health may have shaped the direction of the interviews, with her insights and probing questions encouraging deeper exploration of topics related to digital technology use in health care. Overall, these factors likely contributed to a data collection process that was more attuned to the study setting, while also necessitating reflexivity to account for potential influences on participants’ responses.

### Ethical Considerations

Full ethics approval was granted for this research study by the St. Vincent’s University Hospital Research Ethics Committee (reference number: RS23-023). Explicit consent was sought from participants for the collection and processing of their data. Data processing agreements are in place to ensure that personal data are processed as is necessary to achieve the objective of the health research and to ensure that data shall not be processed in such a way that might cause damage or distress to the participants. St. Vincent’s University Hospital and University College Dublin are joint data controllers for the study. This study complies with the General Data Protection Regulation. Study results are being presented at medical conferences and disseminated via peer-reviewed journals.

## Results

### Overview

**Figure 1.**
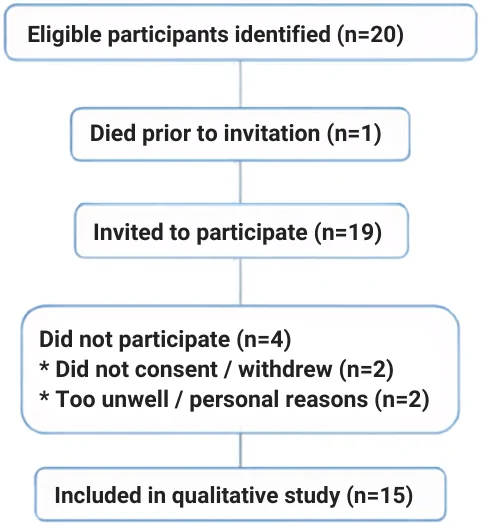
Predicting Outcomes using Digital Technology in Interstitial Lung Disease qualitative study recruitment flowchart.

In total, 15 participants completed the qualitative study (Figure 1).

Demographics of study participants are presented in Table 2. Participants were 80% (12/15) male, 86% (13/15) Caucasian, with a mean age of 62 (SD 16.3) years. This demographic is very similar to the overall PRODIGY-ILD dataset, where participants were 80% (16/20) male, 90% (18/20) Caucasian, with a mean age of 62.5 (SD 14.8) years. Patient-reported outcome measure (PROM) completion was high over the first 12 months (overall adherence >99%). Smartwatch wear time was similarly high, with a median wear time of approximately 96% (IQR 95.9%-99.5%) over 12 months, indicating sustained engagement with the digital monitoring protocol (Multimedia Appendix 4).

**Figure 2.**
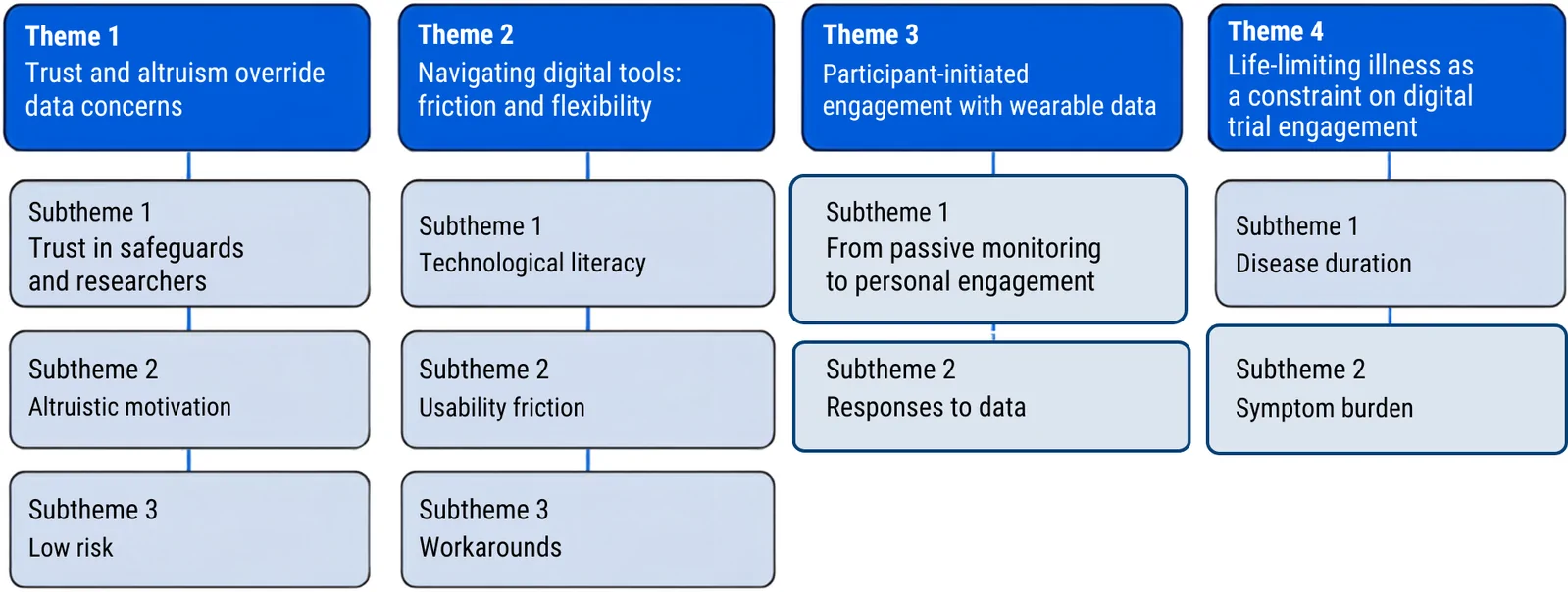
Themes and subthemes from qualitative analysis.

Our findings produced 4 main themes with subthemes (Figure 2). NVivo parent and child codes for each theme are available in Multimedia Appendix 3.

**Table 2.** Participant demographics (N=15).

Variable	Value
Age (years), mean (SD)	62 (16.3)
Gender, n (%)	
Men	12 (80)
Women	3 (20)
Ethnicity, n (%)	
Caucasian	13 (86)
Asian	1 (7)
Mixed	1 (7)
ILD^a^ subtype, n (%)	
IPF^b^	4 (27)
CPFE^c^	3 (20)
CTD-ILD^d^	3 (20)
HP^e^	3 (20)
Fibrotic sarcoidosis	2 (13)
Time since diagnosis (years), median (IQR)	4.5 (2.0‐8.0)
Supplemental oxygen, n (%)	4 (40)
Comorbidities, n (%)	
Cardiovascular	11 (73)
Metabolic	9 (60)
Respiratory	4 (27)
Gastrointestinal	4 (27)
Rheumatological	3 (20)
Malignancy	1 (7)

a ILD: interstitial lung disease.

b IPF: idiopathic pulmonary fibrosis.

c CPFE: combined pulmonary fibrosis and emphysema.

d CTD-ILD: connective tissue disease-associated interstitial lung disease.

e HP: hypersensitivity pneumonitis.

### Theme 1: Trust and Altruism Override Data Concerns

#### Reflexive Thematic Concept

In the context of digital surveillance within clinical trials, participants’ decisions to share personal health data are not solely determined by individual privacy concerns, but are influenced by confidence in research teams, ethical frameworks, and the belief that their contributions have meaningful impact. This analysis synthesizes 3 core subthemes: trust in safeguards and researchers, altruistic motivation, and low perceived sensitivity of data.

#### Trust in Safeguards and Researchers

Participants described why they felt comfortable sharing their data, highlighting the importance of transparent communication in building trust. One patient noted:

...I wasn’t worried, because...I knew from talking to yourself, it’s going to be people who require that information…and are going to use it. To see if they can make improvements.

[Patient C]

One patient backed this up by saying:

As I say, you know, I mean…there’s not enough trust in this world…if you’re doing something like this…then you trust the people that are doing it are gonna be…with all the information.

[Patient D]

These sentiments underscore how personal interactions with researchers can directly influence participants’ willingness to share sensitive data.

Participants accepted data sharing within the “safe” frame of health research in a University Hospital environment with project approval by the Ethics committee. One patient commented, “...obviously, you know, you’ve got fairly strong ethics...to consider...” (Patient E). One patient said, “I just thought….It’s [name of university], it’s a…public body, you got to trust people, don’t you?” (Patient F). This participant’s willingness to share data was rooted in the institution’s reputation.

These findings suggest that clear communication regarding clinical trial governance, such as data security and institutional trustworthiness, may be critical for participant recruitment and retention in clinical trials.

#### Altruistic Motivation

Participants’ belief that their data could help others promoted their willingness to share. Participants expressed a variety of altruistic motivations for sharing their health data (Table 3). The responses suggest that participants viewed their data sharing as a way to directly support others facing similar health challenges, demonstrating a strong sense of empathy and desire to make a positive impact. Participants appeared to see their involvement as an opportunity to advance medical research and improve therapeutic options, reflecting a commitment to the broader scientific community. In addition, participants viewed their data sharing as a way to create a legacy of improved care for others, reflecting a strong sense of social responsibility and a desire to contribute to the well-being of future generations.

**Table 3.** Participant altruistic motivations for sharing health data.

Altruistic motivation	Illustrative participant quotations
Helping others directly	Patient F: “...with the watch..., if it’s going to help somebody else [will] wear it.…If it helps collect data, to find,…trends and stuff like that, then that’s good, isn’t it?” Patient G: “Oh I have no problem with that, Oh, God, no not a problem at all. If it helps others. If it helps with, you know what I’m going through, and probably will be going through worse, I suppose. If it helps others I have no problem at all. No problem. No. No why, why would it bother me at all? No. As I say, if it helps someone else, it’s a great thing.” Patient C: “Didn’t bother me…it doesn’t, as I say, it’s not something that I get excited about. I mean I have a progressive illness.…If I can help, by doing, we’ll say, this, then, take as much information as you require. Do you know, that’s my attitude…”
Contributing to research and advancing knowledge	Patient H: “Oh, fine! If it could be of benefit, that’s great! I mean….Because I find it convenient, and I like it….And mainly what I like, too, is the idea that you are...you’re doing this study.” Patient E: “...and if it’ll help you know, to develop, you know, some additional therapies or monitoring with them. That’s a very good thing. So no, I have no issue with it at all.” Patient J: “I’m glad that it is being collected,…If we can help, alleviate problems, for other people isn’t that…only good. If it contributes to knowledge about this disease. I’m in favour of that. Yes.” Patient I: “I would say that they definitely should consider. Joining it, because really, like, interesting.…Because you could help, like, them to see what’s actually going on and make, you know, new medicine or something. To…to help people with the same condition.”
Supporting future generations	Patient D: “Well, no, I mean, it’s…if the research…leaves…a better understanding….And things like that. And it could help future generations, like…” Patient K: “I’d say you’re giving information now that in the future, people could use, hopefully. For the betterment of life, I suppose. For other people’s health. I’d say, we’re here today, and we get all our medicines through other people doing what we’re doing. But they did it years ago. And…they’ve improved our lives. So why shouldn’t we pay back and improve? Maybe our grandchildren’s life, or whatever. With all this data, all this information, and they correlate it all together. You know well, it’s amazing, what you folk can do with it. I haven’t a clue, but, you know.”

Overall, the range of altruistic motivations expressed by participants, whether helping others directly, advancing research, or supporting future generations, highlights an overarching willingness to share health data for the greater good. These authentic participant voices reinforce the importance of recognizing and honoring these motivations in the design and communication of health research studies.

#### Low Risk

Participants described the health data collected by digital devices (such as steps or heart rate) as nonsensitive and therefore were not concerned about who might access it. Comparisons to bank details were common: for instance, one participant who felt largely unconcerned about sharing this kind of information explained, “I can’t see what…anybody could gain out of it, like, it’s not like you’re giving them your bank details and things like that.” This sentiment was given by another participant, who clarified, “And again, if I mean, it’s not information. That’s you know. It’s not my bank account details.” Such comparisons to the sensitivity of banking information emphasize the distinction between health data and sharing perceived high-risk financial details.

For these individuals, the relatively harmless nature of physical activity or biometric data reduced perceived risks; as one participant put it, “It’s useless,” suggesting that the information collected was not “usable by somebody else.” Another participant described their cautious approach to financial data, stating:

No....The less people to have access to my bank details, the better. I mean, I’ll get some apps that come up, and it says, oh, like, I want you to pay this or pay that, and I just…I just…delete them straight away, like...

These views illustrate how perceptions of sensitivity are shaped by the type of data in question.

However, one participant admitted some hypothetical unease:

I’m not sure…what I would think…if I heard somebody was hacking my devices and taking my health data. Because…I’m not sure if there’s anything personal in what the watch is collecting. Sorry, sorry. When I say personal, I mean usable by somebody else.

This response highlights the variability in how participants assess the sensitivity of their data and that, for some, concerns about unauthorized access linger even when the data seem relatively harmless.

This response may reflect varying levels of digital literacy or previous experiences with data breaches, suggesting that educational interventions could influence perceived sensitivity. For many, a low perceived sensitivity facilitated trust and willingness to share, while for one, residual concerns persisted. These findings indicate that researchers must remain attentive to the different perceptions of data sensitivity and tailor engagement strategies accordingly.

Taken together, these findings suggest that participant engagement with digital health trials can be supported by building interpersonal trust, transparent ethical safeguards, and clearly framing the perceived value and safety of the data being collected. Researchers should consider how trial communications, onboarding, and technology framing can promote a sense of contribution without harm.

### Theme 2: Navigating Digital Tools: Friction and Flexibility

#### Reflexive Thematic Concept

Participants’ experiences with wearable technology and its accompanying app were strongly shaped by individual attitudes, digital literacy, device usability, and workaround strategies. While some approached the technology with confidence and curiosity, “I suppose I tried it because I love gadgets…I was never not going to have a smart watch,” others encountered hesitancy and anxiety when facing practical challenges such as charging, syncing, or app lag. The type of smartphone (Android vs Apple) and the need to use unfamiliar or multiple devices created additional friction. One patient shared:

I haven’t used apple things before, so…to be honest, I had…I can hardly make head or tail of the…iPhone, to be honest with you.

[Patient J]

#### Technological Literacy

Literacy played a key role in shaping participants’ engagement with the digital tools. Individuals who were more comfortable with digital devices generally described the technology as intuitive, unobtrusive, and even enjoyable to use. One patient described the watch as being, “a brilliant piece of technology,…I found it so helpful” (Patient C). For these participants, some cited age (Patient L) as an advantage with fewer barriers. Others described overcoming initial hesitations or lack of interest (Patient I).

In contrast, participants with lower technological confidence described themselves as “technophobes” and often relied on family or informal caregivers for setup and troubleshooting. As one patient described, “my daughter set it up for me…knew what the phone was all about. So, yeah, I got used to it pretty quick” (Patient K). Some participants acknowledged a shift in attitude as the perceived usefulness of the tools became clearer, suggesting that skepticism was not fixed but context-dependent (Patient J).

The varying levels of technological confidence, experiences, and attitudes (Textbox 1) among participants highlight the need for tailored onboarding processes and ongoing support.

Textbox 1. **Participant experiences with technology.**

**Positive experiences**

- Patient L: “To be honest, it was fine for me. I suppose maybe my age helped that. If I’d been older, obviously, it would have been more of a struggle.”
- Patient E: “I’m perfectly happy with the technology. You know, to me, it’s….As technology goes, it’s very easy.”
- Patient I: “I just never really think about it at all, or had the idea to actually use it. But now, since I started using it, I find it really more interesting. How it can track everything.”
- Patient C: “Give it a chance. Use it, because you will find it useful. The technology isn’t scary, and it works. And I found it incredibly helpful in my day-to-day activities.”

**Challenges and scepticism**

- Patient F: “I was always a bit of a technophobe. I tended to think they were a bit of a...a bit some of the things a bit gimmicky, some of the stuff.”
- Patient K: “It was awkward for me because, as I said, I’m totally illiterate when it comes to computers and whatever.”
- Patient M: “I am one of these people that, when I got my mobile phone, I got it for me to use. Not for other people to contact me.”
- Patient J: “I’m not a big fan of technology. I’m not really a fan of it at all, like,….Although I’m very used to the Apple Watch now. I’m a bit of a troglodyte.”

#### Usability Friction

Everyday issues such as remembering to wear the device after showering, keeping it charged, app lag, syncing problems, and confusion over duplicate user profiles frequently disrupted initial adherence. One patient noted:

The only time I don’t wear it is when I take a shower...occasionally I forget to put it back on and again I would defend myself with my age, my issues, and being woolly headed sometimes.

[Patient E]

One patient commented:

my biggest problem was charging when I was having a shower and remembering to put it back on…until you got used to it, you’d forget the odd day to put it back on.

[Patient F]

One patient appreciated aspects such as device comfort and battery life:

It’s very comfy, very usable…the battery lasts for ages. It’s good, I was a bit worried about it, but it lasts a day or two.

[Patient F]

Technical issues with the app were also a concern; some struggled with questionnaires and app responsiveness, as one patient described:

I do find it a bit laggy at times...you do the first one, and then if you’re kind of too quick doing the second one, it doesn’t take, and you have to go back in and do it.

[Patient L]

One patient described problems with confusion over user profiles:

Obviously, I think some days I’ve forgotten to do the questionnaires, although there’s been a couple of times when that’s happened, where I’m nearly convinced that I did answer the questionnaires, and then I’m wondering, did I somehow use the wrong profile.

[Patient E]

Participants expressed the need for more flexibility in device and platform choices to better fit their routines and preferences. One patient described:

An Android user, like me, who kept two separate phones, wasn’t integrating it into their lives properly….To use them properly, you have to be able to integrate them into your life, and they become not just a data collector...

[Patient M]

These technical challenges highlight the importance of user-centered, flexible digital health designs.

#### Workarounds

To overcome initial technological barriers, participants used social support and developed routines. One patient gets family support:

I would need some assistance, yes, because I’m not...I’m not too good on this technology....Yes well [my son] goes over things with me there that are on....he comes out every week....He looks over things, and tells me about this or that....He’s well up on all of that.

[Patient B]

More confident users like Patient E independently sought solutions:

so like I say, it just took me a while to you know, to work around the format of the watch, because again, that was different to the watch I’d had before. But I mean, it’s, you know, it’s a graphical interface. It’s not that difficult to you know, to work through. And you can always Google it if you need to. So yeah, no, I don’t think I had too many issues with that.

One patient described developing routines to efficiently complete required tasks:

Because it tends to be just the two questions, the breathing and the coughing. You know, it only takes 30 seconds to do it. So I tend, as soon as I get the message come through, I’ll tend to do it straight away. But the, the one where it could be, sort of, 19 questions, or whatever. You know, I tend to sort of sit down for an hour, and then go through them properly, and...try and be as accurate as I can. No, it’s fairly straightforward, and, you know, there’s nothing complicated about it.

[Patient D]

One patient took a pragmatic approach to device notifications:

...when I’m on my bike. It kind of pings the watch. And it says something like. I see you’re working out. Or your cycling, or something like that. And, uh, I tend to ignore it.…I just click it back to what it was….I’m just…wearing it as a watch.

[Patient F]

In terms of charging routines, one patient describes:

my biggest problem was charging when I was having a shower and remembering to put it back on,….[Now I]...put it back on straight away. You know, as soon as you come out and dry your hair.

[Patient L]

These varied adaptation strategies demonstrated resilience and resourcefulness, regardless of initial technological proficiency.

Workarounds, whether social (family assistance) or individual (routine-building), illustrate the importance of both personal and environmental resources in successful adoption. Informal caregiver involvement becomes an unplanned implementation mechanism; trials may need to recognize and resource this hidden support network.

Feedback on app reminders was mixed: some found them helpful for adherence, others considered them intrusive (on bank holidays), highlighting the importance of customizable engagement strategies.

Designing trials that acknowledge the desire for more flexibility in device and platform choices highlights the importance of user-centered design. Allowing participants to use their own devices (Bring Your Own Devices [BYOD]) and offering customizable engagement strategies can improve trial inclusivity and reduce the burden on participants.

### Theme 3: Participant-Initiated Engagement With Wearable Data

#### Reflexive Thematic Concept

Although the trial design intended passive data collection, participants frequently engaged actively with their wearable data. For many, the device became embedded into daily routines, supporting self-monitoring, emotional reassurance, and in some cases behavior change, with the majority (14/15) expressing intention to continue using the wearable. Participant-initiated engagement revealed both empowering and distressing impacts, underscoring the personal meanings participants assigned to digital data.

#### From Passive Monitoring to Personal Engagement

Passive data collection can become active self-monitoring, altering behavior or anxiety levels. The engagement with wearable data spanned a spectrum from enthusiastic daily use to more passive or intermittent interaction, with most (11/15) individuals noting specific health benefits and increased self-efficacy.

For some, the device remained a passive tool, with limited personal interaction, as one patient reflected:

I’d just go back to my old watch, so…I’m sorry, yeah, it’s not…I should be gushing about it, but I’m not…,

[Patient F]

This illustrates that when data collection is entirely passive and lacks user interaction, engagement may remain minimal.

In contrast, others found significant reassurance and utility in the ability to retrospectively review their health data. One patient shared:

...I find it incredibly useful, and it helps me, and it also helps the fact that…the readings are stored on the iPhone. And you can go back and look through them. Plus, it averages out your results over a week, a month, 6 months. And you can start to see if you’re…going up, down, or you’re remaining stable? So, from that point of view, it’s absolutely super.

[Patient C]

This highlights how long-term data tracking and the ability to observe trends can provide participants with a sense of control and reassurance.

For some, the wearable device offered emotional support and comfort. One expressed, “I use it I feel it…it is a comfort to have it,” suggesting that the device became more than a data collector—it provided a sense of security (Patient B). One patient went further, describing the device as integral to her sense of self: “If I don’t wear it, is it my….It’s…it’s my…myself is not complete” (Patient N). This sentiment underscores how, for certain individuals, the wearable became an extension of their identity.

Curiosity and discovery also motivated ongoing engagement for some participants. One patient remarked:

I find it, like, really interesting, because I never really thought of. How much an apple watch can actually do in the health app? And I just find it really interesting.

[Patient I]

This sense of discovery fostered a positive attitude towards the technology and encouraged regular use.

Others described how the device became embedded in their daily routines, supporting proactive self-monitoring and health management. One patient explained:

I would generally look at it in the morning for the oxygen overnight, and then at night, I would check everything, so I would check heart rate for the day. Oxygen saturation, steps walked,…Oh, in the morning, I would check…sleep and oxygen saturation,...then in the evening, everything.

[Patient L]

This routine use demonstrates how wearables can facilitate regular health checks and empower participants to take an active role in managing their condition.

Collectively, these narratives illustrate the diverse ways in which participants engaged with wearable technology—ranging from limited interaction to deep integration into daily life. The emotional and practical significance of these devices varied, but for many, they provided reassurance, comfort, curiosity, and a means to monitor and manage their health more effectively. These insights highlight the importance of considering individual preferences and experiences when designing digital health interventions for people living with chronic conditions.

#### Responses to Data

Participants described both comfort and anxiety in response to the data. For some, it provided reassurance and control; for others, it increased worry, especially when data confirmed decline. Some participants changed behaviors based on data while others did not.

Participants described how wearable device data influenced their understanding and management of their health. For example, one patient reflected:

It’s great, because you notice, right, I’m not imagining it. It is there, and it’s something, like I was able to say. Like, weirdly enough. Whatever the hell it was. Tuesdays were terrible for AFib. All my episodes have either been on a Monday night, or Tuesday morning.

[Patient C]

This demonstrates how device data enabled validation of symptoms and identification of patterns, enhancing self-understanding.

One patient shared:

Yes, it tells me how many hours I slept. Which was a good thing, because then I could justify why I was tired.

[Patient K]

Here, the objective data supported self-justification and explanation of symptoms, reinforcing the device’s utility in daily life.

One patient explained:

I can sit down and relax and watch it and see if the heart rate starts to go back down. Then I get settled, more settled then you know.

[Patient G]

This illustrates how real-time feedback from the device was used to guide self-regulation and coping strategies.

One patient noted:

Yeah. I tried to look at it almost, like, every single day. It’s kind of like a routine I like to see if it’s, like, normal, high, or low.

[Patient I]

For Patient I and others, daily engagement with wearable metrics became a habitual part of life.

However, not all experiences were reassuring. One patient said:

It definitely didn’t reassure me, and it made me the opposite, because I could...I knew I was...I knew I was starting to struggle more, and I could see, probably, it was progressing, and I suppose the stats show that, you know, when I, when I checked them, I could see the decline and everything.

[Patient L]

For some, data indicating deterioration contributed to increased anxiety, showing the nuanced emotional impact of continuous tracking.

A patient described:

Because I have got used to it now...I’m watching the…you know, steps and that. Like, the Fitbit aspect of it. Again, it’s not something I’d be obsessive about, but it’s just interesting to see.

[Patient J]

This suggests that integrated tracking features can foster ongoing, non-intrusive engagement without leading to obsession.

Finally, one patient commented:

I don’t have any little goals or anything, I just...observe what’s happening with it, and that’s….Okay, no, it’s convenient, and….Yes, I realize that it’s…that it’s there for the statistical records, and that you’re interested in the nighttime heart rates and all these things. Of course, I’m interested in it, too. I have a personal interest in it.

[Patient H]

This reflects how some participants engaged with data in a more observational, less goal-oriented manner, highlighting the diversity of usage styles.

The wearable device transitioned from a passive study requirement to a routine aspect of daily life. This integration facilitated regular health checks, promoted new routines, and, for some, prompted behavior change, an unintended consequence. This illustrates how wearables were embedded into morning and evening routines, supporting self-monitoring and proactive health management. Continuous monitoring can have both reassuring and anxiety-inducing effects on participants. Providing clear communication about the purpose and benefits of data collection, along with emotional support resources, can help mitigate negative emotional impacts.

### Theme 4: Life-Limiting Illness as a Constraint on Digital Trial Participation

#### Reflexive Thematic Concept

Participants’ engagement with digital tools was shaped by the length of time they have had the diagnosis and ongoing demands of managing chronic and progressive illness. Coping with fatigue, breathlessness, medication routines, and oxygen setup already demands much of patients before introducing additional digital devices.

#### Disease Duration Might Foster Routine Management or Digital Fatigue

Participants reported a wide range of diagnostic journeys (Multimedia Appendix 5), from recent confirmations to decades-long histories of illness. Some described protracted periods of misdiagnosis or uncertainty, while others experienced abrupt transitions from health to chronic disease management.

Longer disease duration may foster routine management strategies but could also lead to “digital fatigue,” a diminished motivation to engage with new technologies due to the cumulative demands of ongoing self-monitoring. Conversely, those with recent diagnoses sometimes reported uncertainty and a steeper learning curve in adopting digital tools. Notably, a dropout from the study who did not consent to be interviewed had been diagnosed a year previously, citing anxiety with using digital tools. In both cases, the context of diagnosis duration shaped the readiness and capacity for digital trial participation.

#### Symptom Burden May Shape Willingness or Capacity to Use Technology

Participants described how illness-imposed restrictions fundamentally altered their daily routines, social activities, and independence:

If I want to do anything, I’ve got to think oxygen. If I don’t, I’ll just get caught short. And I’m like a fish out of water, I’m floundering.

Reliance on supplemental oxygen was a defining feature for many, shaping both physical mobility and daily planning: “I wake up in the morning, and before I go to the toilet or do anything, then the oxygen has to go on....” The logistics of managing oxygen supplies and equipment often required advance planning and adaptation, leaving less flexibility for unscheduled or spontaneous engagement with digital trial tools. One participant had so much to take with them for a trip they didn’t bring the study phone:

I went up to [the city] Saturday, stayed overnight, came back yesterday evening. I didn’t bring my phone with me, you know? So that’s the only thing…be inclined to forget…the phone.

Another spoke about having enough to do when faced with throat cancer to focus on wearing the watch and charging it, “No, I was more concerned about the cancer in my throat. I didn’t think of the watch. You know?” This participant’s watch wear time completely stopped when faced with cancer.

Cough varied in frequency and severity, with some experiencing persistent symptoms and others only occasional bouts, while for others it seemed irrelevant:

…I’m keeping in mind what I’ve been putting in on the…on the MyCap as well, and I know that. Most of the time, it’s been…pretty stable, like….The last two weeks now...my…cough has been getting a little bit worse, because today now, when I’m coughing. It’s actually painful in my chest. Now, I don’t know whether I have picked up an infection or something. But, as I say, I’ll sort of wait and see.

All the questionnaires that you’ve been giving me about coughing, I never understood them….But since about the 2nd of [Month] I am now getting coughing fits....

I get two…questionnaires a week. And one of them is about my cough…I don’t really have a cough…doesn’t seem that relevant to me

Fatigue was a prominent and often unpredictable barrier, affecting both physical stamina and cognitive function: “I have a lot of fatigue….I’m sometimes woolly headed,” “There’s just no energy. I’ve no energy at all,” “I get tired very quickly…it varies from day to day, I mean, some mornings I get up and I feel fine....But then there are other days I wake up, and I’ve just got nothing in the tank.” Fatigue can be a cause of delayed interactions with digital trial tools. The first dropout from the study who declined to be interviewed had been vague in responses to follow-up consistently being late to fill out questionnaires. Perhaps this was due to mental fatigue affecting their focus or articulation. The cognitive effects of fatigue make complex or multistep digital tasks more challenging, underscoring the need for accessible, low-burden digital designs in future trials. These limitations often led to prioritization of essential tasks, with discretionary activities, including digital trial engagement becoming secondary. For some, the cognitive and logistical effort required to interact with apps or devices was an additional burden layered atop daily challenges.

Participants’ experiences of life-limiting illness fundamentally shape their engagement with digital technology study tools. Core symptoms—fatigue, breathlessness, cough—and the practical demands of managing comorbidities and oxygen create capacity and motivation challenges. For example, the participant with throat cancer vividly shows how comorbidities directly interrupted study tasks. Understanding this context is essential for designing digital interventions that are feasible, empathetic, and responsive to the lived experiences of people with chronic, life-limiting conditions. To address these challenges, digital interventions should allow flexible participation schedules and low-effort digital interfaces, ensuring that technology adapts to the needs and capacities of participants rather than imposing additional burdens.

## Discussion

### Principal Findings

Participants described core themes influencing engagement with digital health technologies, including trust, digital literacy, emotional responses to data, and illness-related constraints. This qualitative substudy provides an in-depth examination of how individuals living with fibrotic ILD experience sustained participation in a digitally enabled clinical research program. While digital monitoring is increasingly promoted as a means to enhance data granularity and reduce reliance on clinic-based assessments, our findings demonstrate that engagement with such technologies is not passive or uniform. Instead, participation is actively shaped by trust, digital literacy, emotional responses to data, and the constraints imposed by living with a life-limiting illness.

Continuous digital monitoring has the potential to increase granularity of outcome measurement; however, it may also introduce measurement reactivity, whereby participants alter behavior or reporting in response to being observed [19]. In the context of this study, participants had continuous access to wearable-derived data via the Apple Health app (Apple Inc), enabling interaction with trial data beyond the boundaries of the study protocol. While prior work has focused on feasibility and validation of digital end points, our findings highlight how access to consumer platforms such as Apple Health enables participant-initiated use of trial data, introducing behavioral adaptation and potential cointervention [20-22]. These processes may influence activity patterns, symptom reporting, and disease perception, with implications for the validity and interpretation of trial outcomes. Recognizing and characterizing these mechanisms is essential for the design and interpretation of future digital and hybrid trials [23].

Participants interviewed between 8 and 14 months into the planned 3-year study found digital monitoring acceptable, which aligns with emerging evidence showing high feasibility of home monitoring in ILD and other chronic respiratory diseases [24,25]. High PROM adherence (>99%), alongside high smartwatch wear times (median 96%, IQR 95.9%-99.5) in the first 12 months of PRODIGY-ILD, reinforces the feasibility of sustained digital data collection, an essential requirement for longitudinal outcomes. For clinical trials, this suggests that integrating wearables and regular ePROMs is likely to be acceptable to most participants, supporting their inclusion as trial end points or exploratory biomarkers. However, the context provided by qualitative interviews also highlights that certain factors such as comorbidities can limit willingness or capacity to engage with digital tools over time. For some participants, illness-related challenges led to reduced engagement or prioritization of digital trial activities, underscoring the importance of designing flexible, low-burden interventions that adapt to fluctuating participant capacity and lived experience. This suggests that while overall feasibility is high, successful implementation requires ongoing attention to accessibility, participant support, and the minimization of additional burdens.

The qualitative analysis of participant experiences in the PRODIGY-ILD study reveals a number of implications for the design and conduct of clinical trials that incorporate digital monitoring tools. Our 4 themes: trust and altruism, navigating digital tools, adoption of technology, and the impact of life-limiting illness offer insights into how clinical trials can be refined to improve recruitment, adherence, data quality, and participant well-being.

Participants’ willingness to share personal health data was significantly shaped by their trust in research teams, confidence in ethical safeguards, and a strong sense of altruism. Transparent communication about data governance and the institutional reputation of research settings were central to fostering trust. For clinical trials, this highlights the need for clear onboarding processes that articulate data security, privacy protections, and the ethical framework underpinning the study [26,27]. Recognizing and explicitly honoring participants’ altruistic motivations, such as helping others, contributing to research, and supporting future generations, can further strengthen engagement and retention [28,29]. Trial communications should frame participation as a meaningful contribution to the advancement of care, while addressing any residual privacy concerns through ongoing dialogue [30,31].

Participant experiences with digital tools varied widely according to technological literacy, device compatibility, and everyday usability. Those less familiar with technology relied on informal support from family or caregivers, consistent with prior findings that digital literacy strongly shapes engagement and retention in remote studies [32]. Usability friction, such as charging, syncing issues, and app lag, caused frustration and has similarly been recognized as a key barrier to sustained adherence in digital health interventions [33-35]. To mitigate these barriers, clinical trials should incorporate stratified onboarding and support mechanisms tailored to different levels of digital literacy [33-35]. Flexible approaches, such as allowing participants to use their own devices and providing customizable engagement strategies, can reduce burden and improve inclusivity [36]. Recognizing the hidden labor of informal caregivers and resourcing this support where possible may be crucial for sustained adherence, especially among older or less tech-savvy populations [32,35].

The study revealed that participants often engaged actively with their wearable data, leading to both empowering and distressing impacts. While many found reassurance and motivation through self-monitoring, others experienced heightened anxiety when confronted with evidence of disease progression. These findings highlight the importance of developing communication strategies that provide support and emotional resources to help participants understand their data. Trials should carefully consider how much real-time data visibility is appropriate, balancing the benefits of participant engagement against the risk of unintended psychological harm. Structured feedback mechanisms and clear explanations of the purpose and limitations of digital end points should be used to safeguard participant well-being and trial integrity [20,25].

The demands of managing progressive illness, fatigue, breathlessness, medication regimens, and comorbidities significantly shaped participants’ capacity and motivation to engage with digital trial tools [37-39]. For participants with severe comorbidities, the burden of treatment can mean trial activities are deprioritized [37,39]. To accommodate these realities, clinical trials must design digital interventions that are flexible, low-burden, and adaptive to fluctuating participant capacity [22,26]. Features such as adjustable participation schedules, simple interfaces, and options to pause or modify engagement can make trials more feasible and empathetic. Understanding the lived experience of chronic illness should inform all stages of digital trial development, from feasibility testing to ongoing support [16].

### Limitations

There are limitations that should be considered when interpreting the findings of this study. First, the sample size was small (n=15), reflecting the exploratory nature of the work and the intensive, interview-based methodology. While this enabled rich, in-depth accounts, the perspectives captured may not represent the broader population of individuals living with fibrotic ILD. Two participants who dropped out of the study did not consent to being interviewed, which may bias findings toward acceptability of technology use. Participants were also drawn from a single specialist ILD service, which may limit transferability to settings with different patient demographics, service structures, or technology access. Second, several participants relied on informal caregiver support to manage digital trial tasks, such as device setup, troubleshooting, and data entry, which may not be replicable for individuals without such assistance. The role of caregivers, although integral for some participants’ engagement, introduces variability that may influence the feasibility and acceptability of digital tools in more socially or digitally isolated groups. Elements of response bias may have been introduced, as participants could have been influenced by their existing relationship with the researcher, potentially leading them to provide responses they perceived as desirable or expected. Finally, the heterogeneous clinical course of fibrotic ILD, many of which are progressive and may be life-limiting, may have influenced participants’ engagement with the study. Participants described a strong desire to understand their condition, reflecting the scarcity of effective treatments and the uncertainty surrounding disease trajectory. This sense of urgency and investment may have enhanced adherence to digital tools and PROMs beyond what might be expected in conditions with a more stable or treatable course, limiting generalizability to other patient populations.

### Conclusion

Taken together, these findings extend existing literature on the feasibility and acceptability of digital monitoring in ILD by demonstrating that sustained engagement is a dynamic, emotionally mediated process embedded within the broader context of illness, identity, and daily life. While overall adherence in PRODIGY-ILD was high, qualitative insights reveal the effort, adaptation, and negotiation that underpin this success. Embedding qualitative research within digital trials provides critical insight into how technologies are actually used, interpreted, and experienced and is essential for understanding the mechanisms through which digital end points are generated.

In conclusion, successful integration of digital technologies into clinical trials requires more than technical feasibility. It demands a participant-centered approach that builds trust, acknowledges altruistic motivations, accommodates variability in digital literacy, recognizes the role of informal caregivers, and addresses the emotional and practical realities of living with chronic, life-limiting illness. By illuminating these processes, this study contributes to the methodological foundations of digital health–enabled research and offers actionable insights to inform the design, conduct, and interpretation of future digital and hybrid clinical trials.

## Supplementary material

10.2196/91533 Multimedia Appendix 1 Interview script.

10.2196/91533 Multimedia Appendix 2 Consent for qualitative substudy.

10.2196/91533 Multimedia Appendix 3 NVivo parent child codes for each theme.

10.2196/91533 Multimedia Appendix 4 Smartwatch weartime.

10.2196/91533 Multimedia Appendix 5 Participant reported disease duration.

10.2196/91533 Checklist 1 COREQ checklist.

## Abbreviations

BYOD: Bring Your Own Devices
COREQ: Consolidated Criteria for Reporting Qualitative Research
ePROM: electronic patient-reported outcome measures
ILD: interstitial lung disease
PRODIGY-ILD: Predicting Outcomes using Digital Technology in Interstitial Lung Disease
PROM: patient-reported outcome measure
