# Supplementary material for: Patient Experiences of Digital Technology Use in Interstitial Lung Disease (PRODIGY-ILD Study): Qualitative Study Using Reflexive Thematic Analysis
Source: JMIR Hum Factors. 2026 Aug 25;13:e91533. doi: 10.2196/91533 (PMC13505885; doi:10.2196/91533)
Supplement: Supplementary file 1 [file humanfactors-v13-e91533-s006.pdf]

COREQ 32-Item Checklist

Item No.	COREQ Checklist Item	Page/Section in Manuscript
1	Interviewer/facilitator – Who conducted the interview/focus group?	EG Corresponding author
2	Credentials – Interviewer's degrees or qualifications.	MSc Clinical Trials, Graduate Medicine
3	Occupation – Interviewer's occupation at time of study.	PhD Digital technology for clinical trials
4	Gender – Interviewer/facilitator gender.	Female
5	Experience and training – Relevant background/training.	Methods/ Research team and reflexivity
6	Relationship established – Was a relationship established prior to study?	Yes, participants were onboarded to study and supported by EG
7	Participant knowledge of interviewer – What did participants know about the researcher?	Methods/ Research team and reflexivity
8	Interviewer characteristics – Biases, assumptions, reasons for interest in the topic.	Methods/ Research team and reflexivity
9	Methodological orientation – e.g., reflexive thematic analysis.	Methods/ Design and theoretical approach
10	Sampling – How were participants selected?	Methods/Participant recruitment
11	Method of approach – How were participants contacted?	Methods/Participant recruitment
12	Sample size – Number of participants.	Methods/Participant recruitment
13	Non-participation – Number declined/dropped out; reasons.	Methods/Participant
14	Setting of data collection – Location/context of data collection.	Methods/Data Collection
15	Presence of non-participants – Anyone else present during interviews?	Methods/Data Collection
16	Description of sample – Demographics, relevant characteristics.	Results/Table 1
17	Interview guide – Were questions/pilots tested?	Methods/Data Collection
18	Repeat interviews – Number conducted.	None
19	Audio/visual recording – Was it used?	Methods/Data Collection
20	Field notes – Were they made during/after interview?	Methods/Data Collection
21	Duration – Length of interviews/focus groups.	Methods/Data Collection
22	Data saturation – Was it discussed?	Maximum number of participants were recruited
23	Transcripts returned – Were transcripts returned for comment/correction?	Methods/Data collection

24	Number of data coders – How many coders analysed the data?	One coder with two supervisors
25	Description of coding tree – Provide outline/diagram.	Supplementary data
26	Derivation of themes – Pre-set or emergent themes?	Emergent
27	Software – Was software (e.g., NVivo) used?	NVivo
28	Participant checking – Did participants provide feedback on findings?	No did not want to burden
29	Quotations presented – Were participant quotations used?	Yes
30	Data and findings consistent – Is there consistency between data and conclusions?	Yes
31	Clarity of major themes – Are they clearly described?	Yes
32	Clarity of minor themes – Is there a description of diverse/nuanced cases?	Yes
