# Supplementary material for: Patient Experiences of Digital Technology Use in Interstitial Lung Disease (PRODIGY-ILD Study): Qualitative Study Using Reflexive Thematic Analysis
Source: JMIR Hum Factors. 2026 Aug 25;13:e91533. doi: 10.2196/91533 (PMC13505885; doi:10.2196/91533)
Supplement: Supplementary file 2 [file humanfactors-v13-e91533-s001.docx]

**PRODIGY-ILD Study: Qualitative Interview Template (Digital Tech Sub study)**

**Participant ID:**

**Date:**

**Interviewer:**

**Interview Mode:** (In-person / Phone / Video)

**1. Introductory Script**

“Thank you for agreeing to take part in this interview. As part of the PRODIGY-ILD study, we’re interested in your experiences using the app and Apple Watch since you started the study. Your insights will help us understand how this kind of digital technology works for people living with ILD and how we might improve it for the future. There are no right or wrong answers — we’re just keen to hear your honest opinions. You can pause or stop at any time.”

**2. Background & Context**

- Could you briefly describe your experience living with ILD so far?

*Prompt:* *“How long have you had the diagnosis? Can you take me through a normal day, or a time when your symptoms affected your daily life?”*

- Before joining this study, had you used any health-related apps or wearable technology?

*Prompt: “For example, step counters, smartwatches, or symptom trackers?”*

*What made you try (or avoid) technology like that before this study?*

**3. Onboarding & Initial Impressions**

- Can you tell me about what it was like first getting started with the app and Apple Watch?

*Prompts:*

*- how did you feel about using the technology at the beginning?*

*- what do you remember about the first time/week you used it?*

*- did anything surprise you during setup?*

*- can you describe a time that stands out at the beginning*

**4. Ease of Use & Functionality**

- What has your day-to-day experience been like using the app and Apple Watch?

*Prompts:* *“What parts felt easy or confusing?”* / *“Can you describe a time when it worked well or badly?”*

- How did you find the reminders?

*Prompt:* *“Any reminders that stood out as especially helpful or annoying?”*

- What was your experience wearing and using the Apple Watch?

*Prompt:* *“*Was there a time you chose not to wear it*? why/why not”*

- Any technical difficulties?

*Prompt:* *“How did you solve them, if at all?”*

- Was there anything that particularly worked well or didn’t work?

*Prompt:* *“Any feature you especially liked or disliked?”*

**5. Perceived Usefulness**

- Did you ever look at your data on the app or watch?

*Prompt: “If so, can you tell what you looked at and why?”*

- Did you notice any changes in your activity, symptoms, or routines because of the technology?

*Prompt: “Can you tell me about a specific time when that happened?”*

- Did you learn anything from the data?

*Prompt:* *“Any numbers or trends you found interesting or confusing?”*

- Did seeing your data motivate or reassure you in any way?

*Prompt:* *“Or did it not affect you at all?”*

**6. Impact on Daily Life**

- How did the technology fit into your daily routine?

*Prompt:* *“Morning, night, during activity?”*

- Were there any days or situations where it was difficult to use?

*Prompt:* *“Can you tell me about a time when you left it off .. and why?”*

- Did the technology ever feel burdensome or intrusive?

*Prompt:* *“In what situations?”*

**7. Privacy and Trust**

- How did you feel about your health information being collected?
- Were you concerned about who could see / access your data?

*Prompt:* *“Any specific concerns?”*

- Did you feel you were in control of what was being tracked?

*Prompt:* *“Would you have liked more options to turn things on/off?”*

**8. Suggestions for Improvement**

- What would you change about the app or the watch?

*Prompt:* *“Any feature that would make it easier or more useful?”*

- Is there anything you wish the app/watch could do that it doesn’t currently?
- If you could design the app/watch yourself, what would it look like or do differently?
- Would you continue using this technology after the study ends? Why or why not?

**9. Closing Questions**

- Is there anything else you'd like to share about your experience?
- Any advice you’d give to someone newly joining this study?

**Closing Statement**

“There are no consequences for anything you say; your answers are confidential and only used for research.”

**Interviewer Notes:**

- Observations (e.g., participant mood, context, interruptions):
- Key themes/quotes (to highlight in analysis):
