# Supplementary material for: Patient Experiences of Digital Technology Use in Interstitial Lung Disease (PRODIGY-ILD Study): Qualitative Study Using Reflexive Thematic Analysis
Source: JMIR Hum Factors. 2026 Aug 25;13:e91533. doi: 10.2196/91533 (PMC13505885; doi:10.2196/91533)
Supplement: Supplementary file 3 [file humanfactors-v13-e91533-s002.docx]

**Patient Consent Form Qualitative sub study addition**

**QUALITATIVE COMPONENT**

You are currently participating in the PRODIGY-ILD study, we are now introducing an additional qualitative component to this study to gain deeper insights into patients' experiences and perspectives.

**PURPOSE OF THE QUALITATIVE COMPONENT**

The purpose of this qualitative component is to explore your attitudes, experiences, and perceptions regarding the digital health technology used in the PRODIGY-ILD study. Your insights will help us understand the usability, acceptability, and impact of these technologies on your daily life.

**WHAT** **PARTICIPATION** I**NVOLVES**

If you agree to participate in this qualitative component, you will be invited to take part in a one-on-one interview lasting approximately 30 minutes. The interview will be conducted at a time and place convenient for you, either in person or via a secure video conferencing platform.

During the interview, we will discuss your experiences with the digital tools provided in the study, including any challenges or benefits you have encountered. With your permission, the interview will be audio-recorded to ensure accurate data collection.

**VOLUNTARY PARTICIPATION**

Your participation in this qualitative component is entirely voluntary. Choosing not to participate will not affect your involvement in the main PRODIGY-ILD study or your medical care. You may withdraw from this component at any time without providing a reason.

**POTENTIAL RISKS AND BENEFITS**

RISKS:

The risks associated with this qualitative component are minimal. Some questions may touch upon personal experiences, which could evoke emotional responses. You are free to skip any questions you are uncomfortable answering.

BENEFITS:

While there may be no direct benefit to you, your participation will contribute valuable information that may improve the design and implementation of digital health tools for ILD patients in the future.

**CONFIDENTIALITY**

All information collected during the interview will be kept confidential. Audio recordings will be securely stored and only accessible to the research team. Transcripts will be anonymized, and any identifying information will be removed. Findings from this component may be published or presented at conferences, but your identity will not be disclosed.
