# Supplementary material for: Patient Experiences of Digital Technology Use in Interstitial Lung Disease (PRODIGY-ILD Study): Qualitative Study Using Reflexive Thematic Analysis
Source: JMIR Hum Factors. 2026 Aug 25;13:e91533. doi: 10.2196/91533 (PMC13505885; doi:10.2196/91533)
Supplement: Supplementary file 4 [file humanfactors-v13-e91533-s003.docx]

**NVivo parent and child codes for each theme**

| **Theme 1** | **Trust and Perceived Research Impact** |
| --- | --- |
| **NVivo Parent code** | **NVivo Child codes** |
| **Trust and Ethical Considerations** | - Advancing Knowledge: Instances where participants discuss how contributing their data or participating in study helps disease research. - Advice to New Participants: Statements where participants offer guidance, reassurance, or recommendations to future participants about sharing data or engaging with the research process. - Attitude to Good Use of Data for Research Purposes: Expressions reflecting participants’ views on the beneficial use of their data within the context of research. - Attitude to Data Being Accessed: Participant reflections on who can access their data and their comfort or concerns regarding such access. - Attitude to Monitoring: Perspectives on the tracking, or surveillance involved in the study, including acceptance or resistance to being monitored as part of research. - Confidence in Ethics: Comments indicating trust in the ethical safeguards, approval processes, or institutional review (such as Ethics Committee approval) implemented to protect participants’ rights and data privacy. |
| **Theme 2** | **Attitudes, Digital Literacy, Usability & Adaptive strategies** |
| **NVivo parent code** | **NVivo Child codes** |
| **Technology Use & Integration** | - Attitude to Technology: Participant’s overall disposition toward using technology, ranging from enthusiasm to scepticism or indifference. Encompasses both general attitudes and specific responses to using digital devices and applications. - Previous use of technology: The type and frequency of participants’ prior engagement with digital devices or apps (e.g., smartphones, wearables), informing their confidence and approach to new technologies - Age Demographic: Participant’s age, captured as a factor influencing technological attitudes and experiences. - Assistance with Technology: Instances where participants relied on external support (e.g., family members) for setup, troubleshooting, or ongoing use. |
| **NVivo parent code** | **NVivo Child codes** |
| **Digital Tools - Apple Watch & MyCap App:** | - Ease of Use of wearable: Participant-reported challenges or ease with operating the wearable device, including wearing, charging, syncing, and or operating the watch. - Ease of use App: interface design, user profile login, syncing issues, or other technical barriers. |
| **NVivo parent code** | **NVivo Child codes** |
| **Technology Integration** | Android versus Apple |
| **Theme 3** | **Unsanctioned engagement with wearable data** |
| **NVivo parent code** | **NVivo Child codes** |
| **Wearable data perceived usefulness** | Engagement with wearable data: Engagement with wearable data: This child code reflects the spectrum of user attitudes ranging from active, interested engagement to more passive or intermittent use—and captures specific reasons for not wearing the device (e.g., discomfort, routine disruptions), which inform our understanding of the broader challenges in sustained technology adoption. |
| **Digital tools: Apple watch & MyCap app** | **NVivo Child codes** |
|  | Use wearable after the study, Psychology of questionnaires**.** App reminders helpful (user feedback on reminders): Focuses on participants’ responses to app-based reminders, such as whether these prompts supported adherence or were perceived as intrusive or useful. |
| **Theme 4** | **Constraints of life-limiting illness** |
| **NVivo parent code** |  |
| **Disease Context** | **NVivo Child codes** |
|  | - Disease diagnosis duration: Refers to the length of time since a patient received their diagnosis. This can affect how comfortable or experienced they are with managing their illness and may influence their willingness or ability to engage with new treatment approaches or digital health tools. - Restrictions on everyday life: Describes the limitations patients experience in their daily routines and activities as a result of their illness. These restrictions often shape independence and social interactions, and can also impact a patient's capacity to participate in digital interventions. - Co-morbidities: Other health conditions that exist alongside the primary chronic disease. The presence of co-morbidities can complicate treatment and self-management, as well as increase the burden of care and affect overall health outcomes. On the flip side if they are interested in using technology to manage a co-morbidity this may facilitate participation. - Supplemental oxygen: The need for additional oxygen support to carry out daily activities. This requirement often indicates severe disease progression and can restrict mobility, social engagement, and the practicalities of using additional devices. - Breathlessness: Difficulty in breathing or shortness of breath, a common and debilitating symptom of chronic respiratory diseases. Breathlessness can limit physical activity and influence how and when patients are able to interact with digital health tools. - Cough: Persistent coughing is another frequent symptom that can disrupt daily life, sleep, and social participation. It may also impact a patient’s comfort and focus when using digital applications. - Fatigue: Ongoing tiredness or lack of energy, often exacerbated by the demands of managing a chronic illness. Fatigue can reduce motivation and stamina for engaging with both routine care and additional digital health interventions. |
