# Supplementary material for: Patient Experiences of Digital Technology Use in Interstitial Lung Disease (PRODIGY-ILD Study): Qualitative Study Using Reflexive Thematic Analysis
Source: JMIR Hum Factors. 2026 Aug 25;13:e91533. doi: 10.2196/91533 (PMC13505885; doi:10.2196/91533)
Supplement: Supplementary file 5 [file humanfactors-v13-e91533-s004.docx]

**Smartwatch wear-time metrics (first year)**

| **Metric** | **Value**  **Median (Interquartile Range)** |
| --- | --- |
| Valid wear days (>10 hours/day) | **358 (310–364) days** |
| Percentage of valid wear days | **98.1% (96.3–99.5)** |
| Total wear hours | **8,005 (7,795–8,470) hours** |
| Percentage of total wear time^a^ | **96.4% (95.9–99.5)** |

^a^Percentage of total wear time was calculated as the proportion of valid wear days (≥10 hours) relative to the total expected follow-up period. All participants were included, with those with shorter follow-up contributing data up to the time of last observation.
