# Supplementary material for: Patient Experiences of Digital Technology Use in Interstitial Lung Disease (PRODIGY-ILD Study): Qualitative Study Using Reflexive Thematic Analysis
Source: JMIR Hum Factors. 2026 Aug 25;13:e91533. doi: 10.2196/91533 (PMC13505885; doi:10.2196/91533)
Supplement: Supplementary file 6 [file humanfactors-v13-e91533-s005.docx]

**Participant reported disease durations**

| Longer Disease Duration > 2 years | Shorter Disease Duration < 2 years |
| --- | --- |
| “I have something called sarcoidosis. I think since 2004, I’m not really sure, but I’ve had the disease and it’s been progressing since then.” | “think maybe a year and a half or so. I'm not quite certain ..So far… I think there's some,… Well, it hasn't been too bad for me,…but I presume it just gets worse and it doesn’t get better. So, you know, I would worry a bit about that.” |
| “I was complaining about a persistent cough, and breathing difficulties, I suppose, as far back as 10 years ago with the GP... it would have been 2021. So it's about four and a half, five years now.” | “It must be a year and a half now, since the diagnosis, roughly, since I was diagnosed it.” |
| “I had an initial diagnosis at the end of 2019 which was actually incorrect. And I got my correct diagnosis, probably around May of 2020...” |  |
| “I was diagnosed, … The first year of COVID,….Until very recently—specifically, in the last 10 days—I managed fairly well since my diagnosis and treatment at [hospital]. The disease progressed slowly, mostly affecting my walking. However, last year, I fell and fractured my spine, which significantly impacted my movement and breathing. That injury has been the most significant effect on my mobility, and recently, something has gone very badly wrong.” |  |
